# New Mechanisms of Flucytosine Resistance in *C*. *glabrata* Unveiled by a Chemogenomics Analysis in *S*. *cerevisiae*

**DOI:** 10.1371/journal.pone.0135110

**Published:** 2015-08-12

**Authors:** Catarina Costa, Andreia Ponte, Pedro Pais, Rui Santos, Mafalda Cavalheiro, Takashi Yaguchi, Hiroji Chibana, Miguel Cacho Teixeira

**Affiliations:** 1 Department of Bioengineering, Instituto Superior Técnico, Universidade de Lisboa, Lisbon, Portugal; 2 iBB—Institute for Bioengineering and Biosciences, Biological Sciences Research Group, Instituto Superior Técnico, Lisboa, Portugal; 3 Medical Mycology Research Center (MMRC), Chiba University, Chiba, Japan; Institute of Biology Valrose, FRANCE

## Abstract

5-Flucytosine is currently used as an antifungal drug in combination therapy, but fungal pathogens are rapidly able to develop resistance against this drug, compromising its therapeutic action. The understanding of the underlying resistance mechanisms is crucial to deal with this problem. In this work, the *S*. *cerevisiae* deletion mutant collection was screened for increased resistance to flucytosine. Through this chemogenomics analysis, 183 genes were found to confer resistance to this antifungal agent. Consistent with its known effect in DNA, RNA and protein synthesis, the most significant Gene Ontology terms over-represented in the list of 5-flucytosine resistance determinants are related to DNA repair, RNA and protein metabolism. Additional functional classes include carbohydrate and nitrogen—particularly arginine—metabolism, lipid metabolism and cell wall remodeling. Based on the results obtained for *S*. *cerevisiae* as a model system, further studies were conducted in the pathogenic yeast *Candida glabrata*. Arginine supplementation was found to relieve the inhibitory effect exerted by 5-flucytosine in *C*. *glabrata*. Lyticase susceptibility was found to increase within the first 30min of 5-flucytosine exposure, suggesting this antifungal drug to act as a cell wall damaging agent. Upon exponential growth resumption in the presence of 5-flucytosine, the cell wall exhibited higher resistance to lyticase, suggesting that cell wall remodeling occurs in response to 5-flucytosine. Additionally, the aquaglyceroporin encoding genes *CgFPS1* and *CgFPS2*, from *C*. *glabrata*, were identified as determinants of 5-flucytosine resistance. *CgFPS1* and *CgFPS2* were found to mediate 5-flucytosine resistance, by decreasing 5-flucytosine accumulation in *C*. *glabrata* cells.

## Introduction

Systemic fungal infections are a problem of increasing clinical significance, in particular for immunocompromised patients, and especially since the extensive use of antifungal drugs, both as treatment and prophylaxis, has led to an increase in the number of infections with intrinsically resistant fungal pathogens [1,2]. This is particularly serious in the case of *Candida glabrata*, the second most common cause of candidemia, found to be considerably more resistant to azoles than other *Candida* species.

The antifungal drug 5-flucytosine is a fluorinated pyrimidine which enters fungal cells through one or more permeases [3,4,5] and is then converted, by cytosine deaminase, to its metabolically active form 5-fluorouracil (5-FU) [3,4,6]. This antifungal drug acts by inhibiting transcription, DNA replication and protein synthesis [3,6]. The specificity of this antimycotic relies on the absence of cytosine deaminase in mammalian cells [4,5]. However, 5-FU is considered toxic, mostly due to the conversion of flucytosine to fluorouracil by gut bacteria [7]. Despite these side-effects, 5-flucytosine it is still used in clinical treatments, mostly in combination with azoles, such as fluconazole, or amphotericin B, for the treatment of *Cryptoccoci* infections [6].

Resistance to 5-flucytosine in clinically relevant *Candida* species develops rapidly in patients under treatment [8]. Resistance can be primary, when it is related with decreased drug uptake of the drug by cytosine permease, encoded by *FCY2* gene, and secondary, when there is limitation in the conversion of 5-flucytosine to 5-FU, or to 5-fluorouridine monophosphate (5-FUMP) by alterations in enzyme cytosine deaminase or uracil phosphoribosyltransferase activity encoded by *FCY1* and *FUR1* genes, respectively [6,9]. Most of these mechanisms have been observed in *C*. *albicans*, but also in other pathogenic *Candida* species [10]. Some studies suggest, however, that molecular mechanisms underlying 5-flucytosine resistance, independent of the Fcy2-Fcy1-Fur1 pathway, may play an important role in this phenomenon [11]. In order to be able to use this very efficient antifungal agent, a global understanding of the mechanisms of yeast resistance towards this drug is required.

In this study, the model yeast *Saccharomyces cerevisiae* was used to identify, at a genome-wide scale, the determinants of resistance to 5-flucytosine. Although a large-scale knockout collection was recently constructed for *Candida glabrata* [12], the genes deleted in this collection only cover around one third of this yeast’s genome, thus leading us to select the *S*. *cerevisiae* disruptome for this study. Based on the identified mechanisms of yeast resistance to 5-flucytosine, the effect of arginine supplementation and cell wall remodeling in 5-flucytosine resistance was inspected in *Candida glabrata*. Furthermore, the suggested role of the *S*. *cerevisiae FPS1* homologues in *C*. *glabrata* was also evaluated.

*FPS1* gene encodes a plasma membrane aquaglyceroporin, whose activity is determined by environmental osmolarity [13]. It was proposed that under hyperosmotic shock Fps1 activity is reduced leading to glycerol accumulation, whereas upon shifting back to hypo-osmotic conditions the Fps1 channel opens to release glycerol and thus relieve turgor pressure [13]. Although its natural substrate seems to be glycerol, Fps1 appears to facilitate the diffusion of toxic compounds across the yeast plasma membrane, including the trivalent metalloids arsenite and antimonite [14], acetic acid [15], boron [16] and ethanol [17], in addition to conferring resistance to numerous unrelated stress agents, such as dithiothreitol, mercaptoethanol, tellurite, tunicamycin, actinomycin D, caffeine, calcofluor white, cycloheximide, doxorubicin and staurosporin, as compiled in the *Saccharomyces* Genome Database (www.yeastgenome.org). Significantly, *FPS1* deletion was shown to disturb the cell redox balance [18] and decrease ergosterol concentration in the yeast plasma membrane [19], an effect likely to interfere with all transmembrane transport systems. Very recently, the *C*. *glabrata* homologs of Fps1, CgFps1 and CgFps2, were found to play a similar physiological role, but also to confer resistance to the antifungal drug caspofungin and to prevent cell wall stress [20]. Based on the chemogenomics data described herein, *CgFPS1 and CgFPS2* were further analysed, in this study, in the context of 5-flucytosine resistance and accumulation in *C*. *glabrata* cells.

## Methods

### Strains and growth media

*Saccharomyces cerevisiae* parental strain BY4741 (*MAT*a, ura3Δ0, leu2Δ0, his3Δ1, met15Δ0) and the derived single deletion mutant collection, with all non-essential genes individually deleted, were obtained from Euroscarf (http://web.uni-frankfurt.de/fb15/mikro/euroscarf/). Cells were cultivated at 30°C, with orbital agitation (250 rpm) in MMB minimal medium, with the following composition (per liter): 1.7 g yeast nitrogen base without amino acids or NH4^+^ (Difco), 20 g glucose (Merck) and 2.65 g (NH4)_2_SO4 (Merck), supplemented with 20 mg/l methionine, 20 mg/l histidine, 60 mg/l leucine, 40 mg/l tryptophan, 30mg lysine and 20 mg/l uracil (all from Sigma). *Candida glabrata* parental strain KUE100 [21] and derived single deletion mutants KUE100_*Δcgfps1* or KUE100_*Δcgfps2*, constructed in this study, as well as the *C*. *glabrata* strains 66032u and 66032u_*Δcgpdr1* [22], kindly provided by Thomas Edlind, from the Department of Microbiology and Immunology, Drexel University, College of Medicine, Philadelphia, PA, were cultivated at 30°C, with orbital agitation (250 rpm) in MMB minimal medium. Solid media contained, in addition to the above-indicated ingredients, 20 g/l agar (Iberagar).

### Genome-wide screening for deletion mutants with altered susceptibility to inhibitory concentrations of the antifungal drug flucytosine

To screen the Euroscarf single-deletion mutant collection for differential susceptibility towards inhibitory concentrations of flucytosine (Sigma), ranging from 0.02 to 0.09 mg/L, the different strains were prepared as described elsewhere [17]. Using a 96-pin replica platter, these cell suspensions were spotted onto the surface of MMB solid medium, supplemented with the amino acid concentrations mentioned above, and supplemented or not with flucytosine. Susceptibility phenotypes were registered after 3–5 days of incubation at 30°C. At least two independent replicates were obtained for each set of mutants and results were, in representative cases, confirmed by spot assays. The eventual over- or under-representation of GO terms associated to our dataset, compared to the yeast genome, was determined using GOToolBox (http://genome.crg.es/GOToolBox/). Enrichment was considered for p-values below 0.01. Gene classification was further conducted according to their description in the Saccharomyces Genome Database (www.yeastgenome.org)

### β-1,3-glucanase susceptibility assay

To monitor structural changes in the cell wall, a lyticase (β-1,3-glucanase, Sigma) susceptibility assay was conducted as described before [23]. *S*. *cerevisiae* cells were grown in MMB minimal medium, in the presence of 0.7 mg/l of flucytosine, and harvested following 0h or 30min of cell incubation, during the period of early adaptation to stress, and at the exponential growth phase, when the standardized OD_600nm_ of 1.0±0.1 was attained. For *C*. *glabrata* cells, a concentration of 0.7 mg/l of 5-flucytosine was used and exponential growth phase cells were harvested when an OD_600nm_ of 2.0±0.2 was attained. The harvested cells were washed with distilled water and resuspended in 0.1mM sodium phosphate buffer (pH 7). After the addition of 10μg/ml lyticase, cell lysis was monitored by measuring the percent decrease of the initial OD_600nm_ of the cell suspensions. Statistical analysis of the results was performed using analysis of variance, and differences were considered statistically significant for P values <0.05.

### Disruption of the CgFPS1 and CgFPS2 genes

The deletion of the *C*. *glabrata FPS1* and *FPS2* genes (ORFs *CAGL0C03267g* and *CAGL0E03894g*, respectively, according to the Candida Genome Database– www.candidagenome.org) was carried out in the parental strain KUE100, using the method described by Ueno et al. [24]. The target genes *CgFPS1* and *CgFPS2* were replaced by a DNA cassette including the *CgHIS3* gene, through homologous recombination. The replacement cassette was prepared by PCR using the primers 5´- AATCAATAAAAAACAATCTAGACTGATACTATTCTGATACTAAAGTATAACAAAAAGGCCGCTGATCACG-3´and 5’- CAGAAGCACATTGAGGTATACTATCCATGGGCATGACGATTCGTTCCGTTTATTCACATCGTGAGGCTGG-3, for the *CgFPS1* gene, and the primers 5´- TACTACTTTTCTTTGAAGAATAATATACTACACTTTGAGACTCCAGCTTGACAAAGGGCCGCTGATCACG-3´and 5’- GTGGAGTGGTAACAATTCATGTAAACTCACATTTACTTTATGAGTAAGAGACCCTACATCGTGAGGCTGG-3, for the *CgFPS2* gene. The pHIS906 plasmid including *CgHIS3* was used as a template and transformation was performed as described previously [21]. Recombination locus and gene deletion were verified by PCR using the following pairs of primers: 5’- CCAAAAATCCCAATGCTATG-3’ and 5’-ATCGTTGTTATTACCGTAG-3’; and 5’-CTATGAGTGACATCCTGGG-3’ and 5’-GACTGTTGGTGTTTGTGG-3’, respectively (S1 Fig).

### Cloning of the *C*. *glabrata CgFPS1* gene (ORF *CAGL0C03267g*)

The pGREG576 plasmid from the Drag & Drop collection [25] was used to clone and express the *C*. *glabrata* ORF *CAGL0C03267g* in *S*. *cerevisiae*, as described before for other heterologous genes [26,27,28]. pGREG576 was acquired from Euroscarf and contains a galactose inducible promoter (*GAL1*), the yeast selectable marker *URA3* and the *GFP* gene, encoding a Green Fluorescent Protein (GFPS65T), which allows monitoring of the expression and subcellular localization of the cloned fusion protein. *CAGL0C03267g* DNA was generated by PCR, using genomic DNA extracted from the sequenced CBS138 *C*. *glabrata* strain, and the following specific primers: 5’—GAATTCGATATCAAGCTTATCGATACCGTCGACA *ATGGAATCTATTCATGATGCTA*—3’ and 5’—GCGTGACATAACTAATTACATGACTCGAGGTCGAC *CTAATATGACACCTTCTCATCA—*3’. The designed primers contain, besides a region with homology to the first 22 and last 22 nucleotides of the *CAGL0C03267g* coding region (italic), nucleotide sequences with homology to the cloning site flanking regions of the pGREG576 vector (underlined). The amplified fragment was co-transformed into the parental *S*. *cerevisiae* strain BY4741 with the pGREG576 vector, previously cut with the restriction enzyme SalI, to obtain the pGREG576_*CgFPS1* plasmid. The recombinant plasmid pGREG576_*CgFPS1* was obtained through homologous recombination in *S*. *cerevisiae* and verified by DNA sequencing.

### Flucytosine susceptibility assays

The susceptibility of the *C*. *glabrata* parental strain KUE100 was compared to that of the derived *Δcgfps1* and *Δcgfps2* deletion mutants, based on growth in solid media. Cell suspensions used to inoculate the agar plates were mid-exponential cells grown in basal MMB medium until culture OD_600nm_ = 0.4±0.02 was reached, and then diluted in sterile water to obtain suspensions with OD_600nm_ = 0.05±0.005. These cell suspensions and subsequent dilutions (1:5; 1:25) were applied as 4μl spots onto the surface of solid MMB medium, supplemented with adequate flucytosine concentrations, from 0.01 to 0.03 mg/L. The same approach was also used to test the effect of *CgFPS1* and *CgFPS2* deletion in the resistance to the cell wall stress agent Calcofluor White (Sigma).

The susceptibility of the parental *S*. *cerevisiae* strain BY4741 towards toxic concentrations of the selected drugs was also compared to that of the deletion mutant BY4741_*Δfps1* by spot assays. The ability of *CgFPS1* gene expression to complement the susceptibility phenotype exhibited by the BY4741_*Δfps1* single deletion mutants was also examined, using the pGREG576_*CgFPS1* plasmid in which *CgFPS1* is expressed under the *GAL1* promoter. *S*. *cerevisiae* cell suspensions used to inoculate the agar plates were mid-exponential cells grown in basal MM4-U medium, containing 0,5% glucose and 0,1% galactose, until culture OD_600nm_ = 0.4±0.02 was reached and then diluted in sterile water to obtain suspensions with OD_600nm_ = 0.05±0.005. These cell suspensions and subsequent dilutions (1:5; 1:25) were applied as 4μl spots onto the surface of solid MM4-U medium, containing 0,1% glucose and 1% galactose, supplemented with growth inhibitory chemical stress concentrations.

To assess the effect of arginine supplementation in *C*. *glabrata* resistance to inhibitory 5-flucytosine concentrations, KUE100 cells were grown in liquid media, supplemented or not with 60mg/L arginine. Cell suspensions used as inocula were mid-exponential cells grown in basal BM medium, supplemented with the above indicated amino acids, until culture OD_600nm_ = 0.4±0.02 was reached. These cells were then harvested by filtration and re-suspended in fresh growth media with an initial OD_600nm_ = 0.05±0.01 and grown at 30°C and 250rpm orbital shaking. Statistical analysis of the results was performed using analysis of variance, and differences were considered statistically significant for P values <0.05.

### CgFps1 sub-cellular localization assessment

The sub-cellular localization of the CgFps1 protein was determined based on the observation of *S*. *cerevisiae* BY4741 cells transformed with the pGREG576-*CgFPS1* plasmid. These cells express the CgFps1_GFP fusion protein, whose localization may be determined using fluorescence microscopy. *S*. *cerevisiae* cell suspensions were prepared by cultivation in MMB-U medium, containing 0,5% glucose and 0,1% galactose, at 30°C, with orbital shaking (250rev/min), until a standard culture OD_600nm_ (Optical Density at 600nm) = 0,4±0,04 was reached. At this point cells were transferred to the same medium containing 0,1% glucose and 1% galactose, to induce protein expression. After 5h of incubation, the distribution of CgFps1_GFP fusion protein in *S*. *cerevisiae* living cells was detected by fluorescence microscopy in a Zeiss Axioplan microscope (Carl Zeiss MicroImaging), using excitation and emission wavelength of 395 and 509nm, respectively. Fluorescence images were captured using a cooled CCD camera (Cool SNAPFX, Roper Scientific Photometrics).

### [^3^H]-flucytosine accumulation assays

[^3^H]-flucytosine transport assays were carried out as described before [26]. To estimate the accumulation of flucytosine (Intracellular/Extracellular [^3^H]- flucytosine) in *C*. *glabrata* cells, the parental strain KUE100 and the mutant strains *Δcgfps1* and *Δcgfps2* were grown in MMB medium till mid-exponential phase and harvested by filtration. Cells were washed and resuspended in TM buffer [0.1 M MES (Sigma), 41 mM Tris (Sigma) adjusted to pH 4.5 with HCl], with 2% glucose, to obtain dense cell suspensions [OD_600nm_ = 5.0 ± 0.2, equivalent to approximately 2.2 mg (dry weight) ml^-1^]. After 5 minutes incubation at 30°C, with agitation (150 rev/min), 0.1 μM of [^3^H]- flucytosine (ICN; 37 MBq/ml) and 100μM of unlabelled flucytosine were added to the cell suspensions. Incubation proceeded for an additional period of 30min. The intracellular accumulation of labeled flucytosine was followed by filtering 200 μl of cell suspension, at adequate time intervals, through pre-wetted glass microfiber filters (Whatman GF/C). The filters were washed with ice-cold TM and the radioactivity remaining in the cells, that were retained in the filter, measured in a Beckman LS 5000TD scintillation counter. Extracelular [^3^H]-flucytosine was estimated at each time point, by radioactivity assessment of 50 μl of the growth culture supernatant. Non-specific [^3^H]-flucytosine adsorption to the filters and to the cells (less than 5% of the total radioactivity) was assessed and taken into consideration. To calculate the intracellular concentration of labelled flucytosine, the internal cell volume (Vi) of the exponential cells, grown in the absence of drug and used for accumulation assays, was considered constant and equal to 2.5 μl (mg dry weight)^-1^ [29]. Statistical analysis of the results was performed using analysis of variance, and differences were considered statistically significant for P values <0.05.

### *CgFPS1* and *CgFPS2* expression measurements

The levels of *CgFPS1* and *CgFPS2* transcripts were assessed by real-time PCR. Synthesis of cDNA for real time RT-PCR experiments, from total RNA samples, was performed using the MultiscribeTM reverse transcriptase kit (Applied Biosystems) and the 7500 RT-PCR Thermal Cycler Block (Applied Biosystems), following the manufacturer’s instructions. The subsequent RT-PCR step was carried out using SYBR Green reagents. Primers for the amplification of the *CgFPS1*, *CgFPS2* and *CgACT1* cDNA were designed using Primer Express Software (Applied Biosystems) and are 5’- TGAGTGACATCCTGGGAAAGG -3’ and 5’- GCCGACGACTGTGGTTATCA -3’; 5’- CATTCCAAAGATGGTGGTTTAAGAG -3’ and 5’- GACATTCTCCTGGCCTTGTTTC -3’; and 5’- AGAGCCGTCTTCCCTTCCAT -3’ and 5’- TTGACCCATACCGACCATGA -3’, respectively. The RT-PCR reaction was carried out using a thermal cycler block (7500 Real-Time PCR System—Applied Biosystems). Default parameters established by the manufacturer were used and fluorescence detected by the instrument and registered in an amplification plot (7500 System SDS Software–Applied Biosystems). The *CgACT1* mRNA level was used as an internal control. The relative values obtained for the wild-type strain in control conditions were set as 1 and the remaining values are presented relative to that control. To avoid false positive signals, the absence of non-specific amplification with the chosen primers was confirmed by the generation of a dissociation curve for each pair of primers. Statistical analysis of the results was performed using analysis of variance, and differences were considered statistically significant for P values <0.05.

## Results

### Genome-wide identification of *S*. *cerevisiae* genes conferring 5-flucytosine resistance

The so-called yeast disruptome, a collection of around 5000 deletion mutants devoid of each non-essential *S*. *cerevisiae* gene, was used to identify all the genes that confer resistance to 5-flucytosine-induced stress. A gene was considered a determinant of 5-flucytosine resistance when its deletion led to absence of growth in the presence of 0.04 μg/mL of 5-flucytosine after 48h, a condition in which the wild-type parental strain is able to grow. Such a chemogenomic approach allowed the identification of 183 genes that confer resistance to 5-flucytosine in *S*. *cerevisiae*.

**Fig 1:**
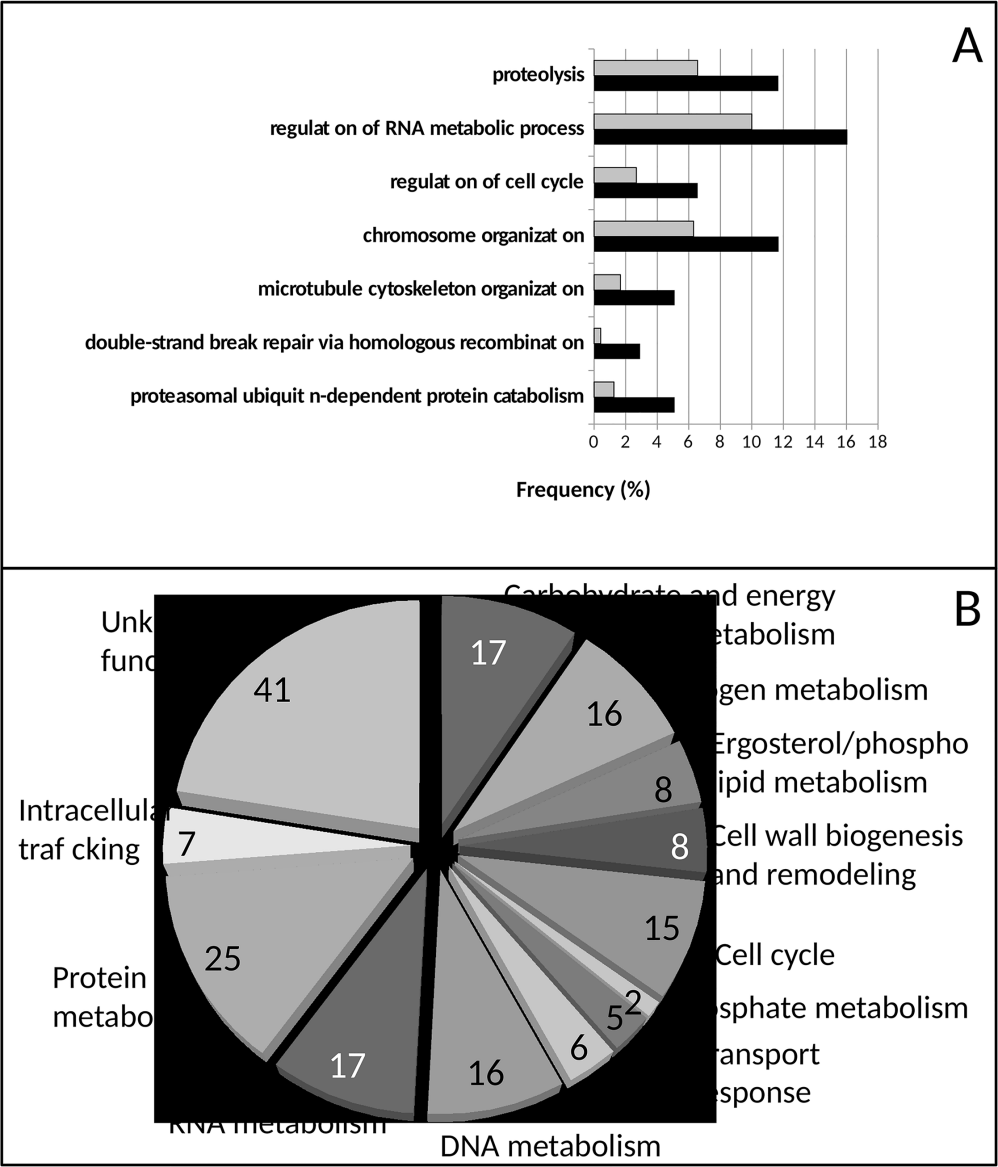
Distribution into functional classes of the 183 determinants of resistance to 5-flucytosine. A) Genes clustered according to the biological process taxonomy of Gene Ontology, using the GoToolBox software (http://genome.crg.es/GOToolBox/). The most highly ranked statistically significant (p-value<0.01) GO terms are displayed. The gene frequency within each class is indicated by the black bars, compared to the frequency registered for the *S*. *cerevisiae* whole genome, indicated by the grey bars, gene frequency being the percentage of the genes in a list associated to a specific GO terms. B) Classification based on the description of gene function registered in the Saccharomyces Genome Database (www.yeastgenome.org). The genes that fall into each class are detailed in supplementary S1 Table.

To obtain a general perspective of the functional distribution of the identified 183 5-flucytosine resistance determinants, GoToolBox was used to identify the GO terms enriched in our dataset, when compared to the genome. These include “proteolysis”, “proteasomal ubiquitin-dependent protein catabolism”, “regulation of RNA metabolic process”, “chromosome organization”, “double-strand break repair via homologous recombination” (Fig 1A). A detailed analysis of the determinants of resistance to 5-flucytosine allowed us to construct S1 Table, in which genes are grouped according to their description in the Saccharomyces Genome Database. A deeper analysis of the most representative groups in the context of 5-flucytosine resistance resulted in the distribution depicted in Fig 1B.

As expected based on the known mode of action of 5-flucytosine, 30 genes related to the metabolism of nucleic acids were found to be determinants of 5-flucytosine resistance (S1 Table). These genes include 13 related to DNA metabolism—5 genes involved in DNA repair, *MRE11*, *RAD55*, *SLM6*, *TOF1* and *XRS2*, and 8 genes involved in chromatin remodeling, *EST3*, *HMI1*, *MFT1*, *NAT4*, *SDC1*, *SNF6*, *SWC3*, *YKU70*—and 17 involved in RNA metabolism—including *CAF40*, *CBC2*, *HTL1*, *IKI1*, *LSM6*, *MSF1*, *NCS6*, *NOT5*, *PIH1*, *PUS1*, *RPA14*, *RPA49*, *SPT8*, *TAN1*, *THP1*, *TRM10*, *ZDS1*.

Twenty-five genes associated to protein metabolism were identified as determinants of 5-flucytosine resistance, highlighting the relevance of the toxic action of 5-flucytosine in protein synthesis and stability (S1 Table). Within this group, it is possible to discriminate 13 ribosome biogenesis-related proteins, 8 proteins required for translation control and 4 proteins associated to protein degradation. Among the ribosome biogenesis proteins found to be required for 5-flucytosine resistance, 4 are specifically related to mitochondrial ribosomes, Mrpl3, Mrpl51, Rml2, Rrf1, whereas the remaining, Bfr1, Rei1, Rpl26b, Rpl2b, Rpl34b, Rpl36b, Rpl43b, Rsa1, Tma23, are involved in cytosolic ribosome formation. The group of protein degradation-associated determinants of 5-flucytosine resistance is composed mostly of proteins with a role in ubiquitination, specifically the ubiquitin-binding component of the Rsp5p E3-ubiquitin ligase complex Bul1, the ubiquitin-conjugating enzyme Rad6 and the ubiquitin ligase Tom1. This group further comprises the vacuolar carboxypeptidase Cps1.

Several proteins involved in carbohydrate and energy metabolism were also found to be required for growth under 5-flucytosine stress. Among them glycolytic/gluconeogenic enzymes Pfk1, Tdh3 and Tps3, but also glycolytic/gluconeogenic regulatory proteins, such as Rim15, a glucose-repressible protein kinase that plays a central role in connecting nutrient-induced signalling pathways, Vid28 and Vid30, which control the degradation of fructose-1,6-bisphosphatase, and Tye7, a transcription factor that positively regulates glycolytic genes. Furthermore, 2 proteins involved in mitochondrial function, Mdm31 and Mdm36, and 4 components of the oxidative phosphorylation pathway, Cyc3, Cox10, Atp2 and Atp17, were also found to confer 5-flucytosine resistance. Finally, peroxisome biogenesis and function-related proteins were identified as determinants of 5-flucytosine resistance, including 2 proteins involved in the control of peroxisome size, Pex29 and Pex32, and one 3-ketoacyl-CoA thiolase, that catalyses one of the steps of fatty acid beta-oxidation. The identification of all these carbohydrate and energy metabolism-related proteins as determinants of 5-flucytosine resistance suggests that 5-flucytosine-stress generates a situation of increased energy demand.

### Nitrogen metabolism and, particularly, arginine metabolism are required for 5-flucytosine tolerance

**Fig 2:**
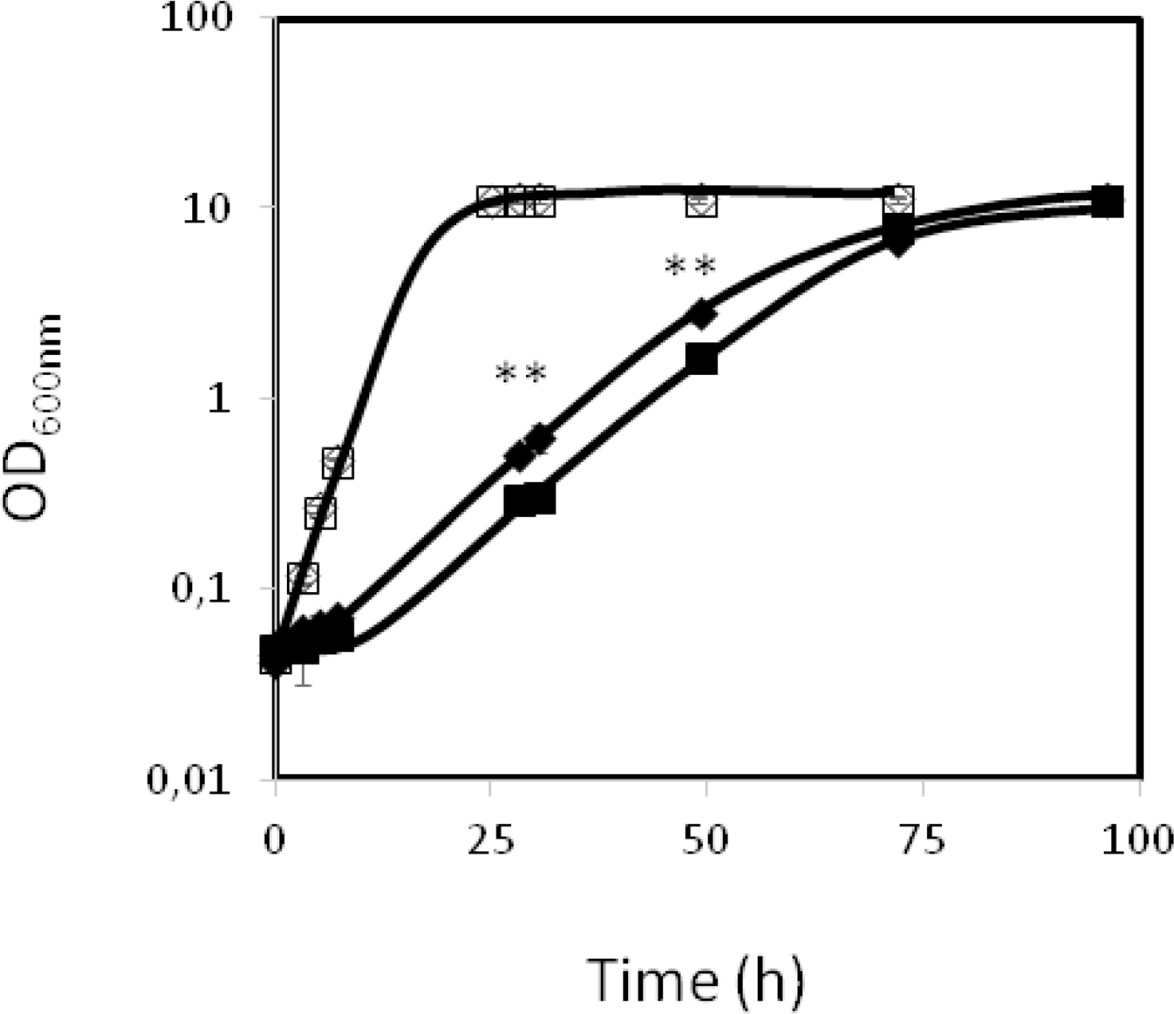
Arginine supplementation decreases the inhibitory effect exerted by 5-flucytosine in *C*. *glabrata*. The growth curves of parental *C*. *glabrata* KUE100 cells in the absence (□,◊) or presence (■,♦) of 0.5 mg/L 5-flucytosine are compared, upon supplementation (◊,♦) or not (□,■) with 60mg/L arginine. The displayed growth curves are the average of three independent experiments. Error bars represent the corresponding standard deviation. **indicates that there is a statistically significant (P<0.01) difference between the OD_600nm_ reached in the presence and absence of arginine, at the indicated times points.

Sixteen genes related to nitrogen metabolism were found to confer 5-flucytosine resistance, including genes involved in the biosynthesis of methionine, *MET2* and *MET7*, proline, *PRO2*, tyrosine/phenylalanine, *ARO7*, and leucine, *TMT1*. Surprisingly, five genes encoding arginine metabolic enzymes, Arg1, Arg3, Arg7, Arg8 and Alp1, were also found to be required for 5-flucytosine tolerance. This observation led us to hypothesize that the concentration of arginine may affect yeast tolerance to 5-flucytosine. This possibility was assessed in the fungal pathogen *Candida glabrata*. The effect of the supplementation of the KUE100 *C*. *glabrata* strain, which is not auxotrophic towards arginine, with 60 mg/l of arginine in the resistance to 0.5 mg/l 5-flucytosine was tested. When exposed to 0.5 mg/l 5-flucytosine, KUE100 cells grown in the absence of arginine exhibited a lag-phase period of around 12h, whereas in the presence of arginine the lag-phase period was reduced to around 8h (Fig 2). Arginine supplementation was found to exert a very subtle effect at the level of the exponential growth rate, in all conditions. Indeed, in control conditions, the population growing in the absence of arginine displayed a growth rate of 0,338±0,031h^-1^, whereas in the presence of arginine the slightly higher growth rate was of 0,352±0,013h^-1^, and the 5-flucytosine-stressed population growing in the absence of arginine exhibited a growth rate of 0,076±0,003h^-1^, while in the presence of arginine the growth rate of the population reached 0,087±0,002h^-1^. The indicated growth rates are the average of three independent growth experiments ± standard deviation.

### Cell wall and lipid metabolism are involved in 5-flucytosine resistance

Despite the fact that 5-flucytosine is not considered to target primarily the plasma membrane or even to affect membrane stability, given its hydrophylicity, eight genes involved in lipid metabolism were found to be required for 5-flucytosine tolerance. These lipid-related genes are the ergosterol biosynthetic genes *ERG3* and *ERG4*, plus six genes that affect the phospholipid composition of the plasma membrane, including the transcription factor encoding gene *MGA2*, that plays a role in the regulation of the desaturase encoding gene *OLE1*, and *OPI1*, which is a negative regulator of phospholipid biosynthesis genes.

**Fig 3:**
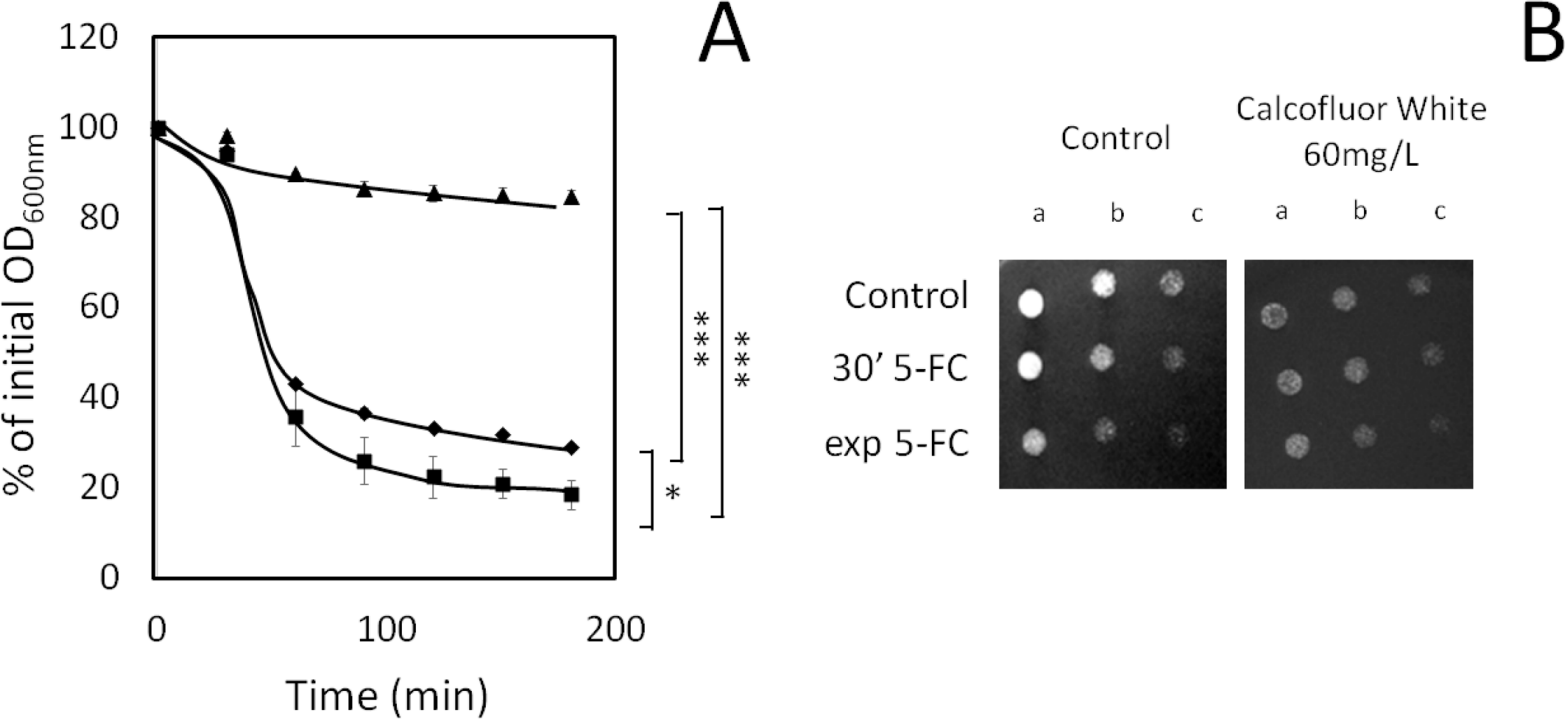
Cell wall damage followed by remodeling occurs during adaptation to 5-flucytosine stress. A) *C*. *glabrata* KUE100 cells exposed to lyticase stress were harvested in the exponential phase of growth in the absence of stress (♦) or upon 30 min of exposure to 0.7 mg/L 5-flucytosine (■), or in the exponential phase of growth reached upon adaptation to 0.7 mg/L 5-flucytosine (▲). The different cell populations were washed with water and resuspended in 0.1M sodium phosphate buffer at pH 7.5. After addition of 20mg/L lyticase, the decrease in the OD_600nm_ of the cell suspension was measured periodically and indicated as a percentage of the initial OD_600nm_. The indicated values are averages of at least three independent experiments. Error bars represent the corresponding standard deviation. * P<0.05; ***P<0.001. B) Comparison, by spot assays, of the susceptibility of *C*. *glabrata* KUE100 cells harvested in the exponential phase of growth in the absence of stress (control) or upon 30 min of exposure to 0.5 mg/L 5-flucytosine (30’ 5-FC), or in the exponential phase of growth reached upon adaptation to 0.5 mg/L 5-flucytosine (exp 5-FC) to Calcofluor White, in MMB agar plates. The inocula were prepared as described in Materials and Methods, and the cell suspensions used to prepare the spots 1:5 (b) and 1:25 (c) dilutions of the cell suspension used for column a. The images are representative of at least three independent experiments.

Additionally, eight cell wall related genes were also found to confer resistance to 5-flucytosine, including *CWP2* and *SED1*, encoding two major constituents of the yeast cell wall, but mostly genes encoding regulators of cell wall composition and remodeling, *CNE1*, *GAS1*, *SCW10*, *SMI1* and *WSC2*. This observation raised the hypothesis that 5-flucytosine may induce cell wall damage and that cell wall remodeling is likely to occur to allow the adaptation of yeast cells to 5-flucytosine. To assess this possibility, two assays were used to test whether or not the cell wall structure is affected by flucytosine action and by the cell response to it: a lyticase susceptibility assay and a Calcofluor White susceptibility test. *C*. *glabrata* cells were seen to exhibit an increase in lyticase (Fig 3A) susceptibility after only 30min of 5-flucytosine exposure, when compared with non-stressed exponentially growing cells. This appears to suggest that 5-flucytosine has a quick deleterious effect at the level of yeast cell wall. Consistent with this idea, upon exponential growth resumption in the presence of 5-flucytosine yeast cells exhibit a reinforced cell wall, even when compared to unstressed yeast cells (Fig 3), possibly resulting from adaptive remodeling. The susceptibility of exponentially growing cells in control conditions, and those exposed for 30’ to 5-flucytosine or until reaching the exponential phase in the presence of 5-flucytosine, to cell wall stress induced by Calcofluor White was additionally evaluated. The results obtained for the control of the Calcofluor White susceptibility assay reveal that the number of viable cells able to grow in solid medium, in control conditions, decreases after exposure to inhibitory concentration of 5-flucytosine (Fig 3B). Under these circumstances, the fact that the same level of growth ability in the presence Calcofluor White is observed for the three cell samples under analysis reveals that indeed the *C*. *glabrata* cells which adapted to growth in the presence of 5-flucytosine are indeed slightly more resistant to cell wall stress than those which were not subjected to stress (Fig 3B).

### CgFps1 and CgFps2 contribute to 5-flucytosine resistance in *C*. *glabrata*

**Fig 4:**
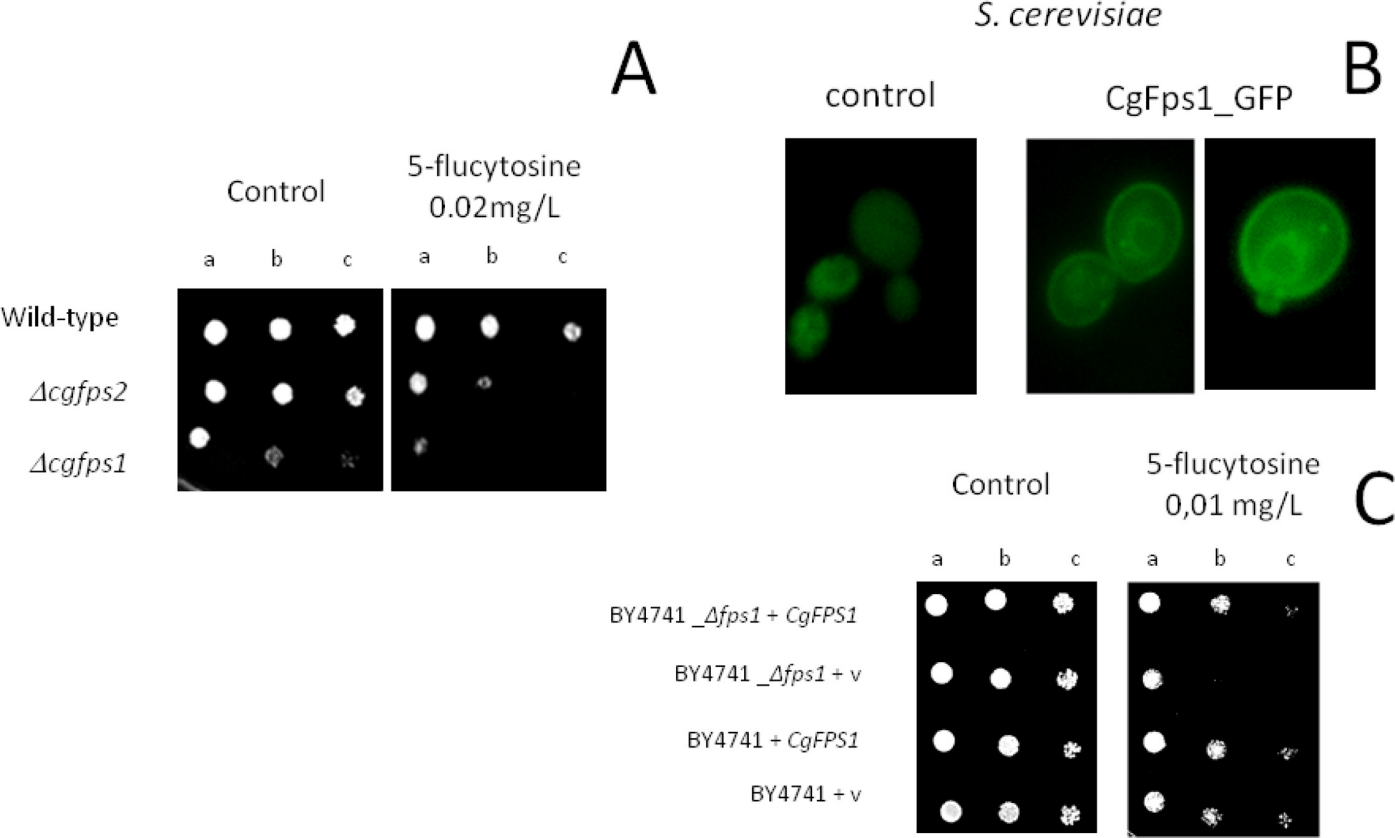
The acquaglyceroporins CgFps1 and CgFps2 confer resistance to 5-flucytosine. **A)** Comparison, by spot assays, of the susceptibility of the *C*. *glabrata* KUE100, KUE100_*Δcgfps1* and KUE100_*Δcgfps2* strains (B) to 5-flucytosine, in MMB agar plates. **B)** Fluorescence of exponential-phase BY4741 *S*. *cerevisiae* cells, harboring the cloning vector pGREG576 (control) or the pGREG576_*CgFPS1* plasmids (CgFps1_GFP), after 5h of galactose-induced recombinant protein expression. **C)** Comparison, by spot assays, of the susceptibility to 5-flucytosine of *S*. *cerevisiae* BY4741 and BY4741_*Δfps1* cells, harboring the cloning vector pGREG576 (v) or the same plasmid expressing the *CgFPS1* gene, in MMB agar plates. In A) and C) the inocula were prepared as described in Materials and Methods, and the cell suspensions used to prepare the spots 1:5 (b) and 1:25 (c) dilutions of the cell suspension used for column a. The images are representative of at least three independent experiments.

The deletion of *S*. *cerevisiae FPS1* gene was found to increase yeast susceptibility to 5-flucytosine, based on the chemogenomic assay described herein. This gene encodes an acquaglyceroporin shown in *S*. *cerevisiae* to be involved in the resistance to several stress agents, as described in the introduction. We, thus, asked ourselves whether the *S*. *cerevisiae* Fps1 protein and its homologs in *C*. *glabrata*, CgFps1 and CgFps2, could be important for yeast resistance to the antifungal drug 5-flucytosine. Indeed, the deletion of *CgFPS2* and especially that of *CgFPS1* was found to increase the susceptibility to 5-flucytosine registered for the parental strain (Fig 4). These genes encode two orthologs of *S*. *cerevisiae FPS1*, encoding plasma membrane acquaglyceroporins required for osmotic stress resistance [20]. Given the role of the Fps proteins in glycerol facilitated diffusion, a possible interconnection between glycerol concentration and 5-flucytosine resistance was hypothesized. To test this hypothesis, the effect of the addition of 1% glycerol to the growth medium, or the effect of having glycerol as the sole carbon source, instead of glucose, in the role of CgFps1 or CgFps2 in *C*. *glabrata* susceptibility to 5-flucytosine was tested. However, no significant effect was observed (results not shown).

To check whether these observations are a direct consequence of gene deletion, the effect of CgFps1 heterologous expression in *S*. *cerevisiae* resistance to 5-flucytosine was assessed. *S*. *cerevisiae* wild-type or *Δfps1* cells harboring the pGREG576_ *CgFPS1* plasmid were grown to mid-exponential phase in minimal medium, and then incubated in the same medium containing 0,1% glucose and 1% galactose, to promote protein over-expression. In these conditions the expression and localization of CgFps1 was analysed through fluorescence microscopy. The CgFps1_GFP fusion was found to be predominantly localized to the cell periphery and, to a lower extent to the endoplasmic reticulum (Fig 4B). Control cells, on the other hand, harboring the pGREG576 cloning vector, displayed a slight and uniform distribution of fluorescence (Fig 4B), similar to what can be observed as the host cells auto-fluorescence. Since CgFps1 is predicted to be an integral membrane protein, these results strongly suggest a plasma membrane localization, similar to what was observed for its *S*. *cerevisiae* homolog Fps1. Based on spot assays, *CgFPS1* was found to confer resistance to 5-flucytosine in *S*. *cerevisiae* (Fig 4B). Significantly, CgFps1 expression appears to complement the 5-flucytosine susceptibility phenotype exhibited by the deletion of its *S*. *cerevisiae* homologue Fps1 (Fig 4B).

**Fig 5:**
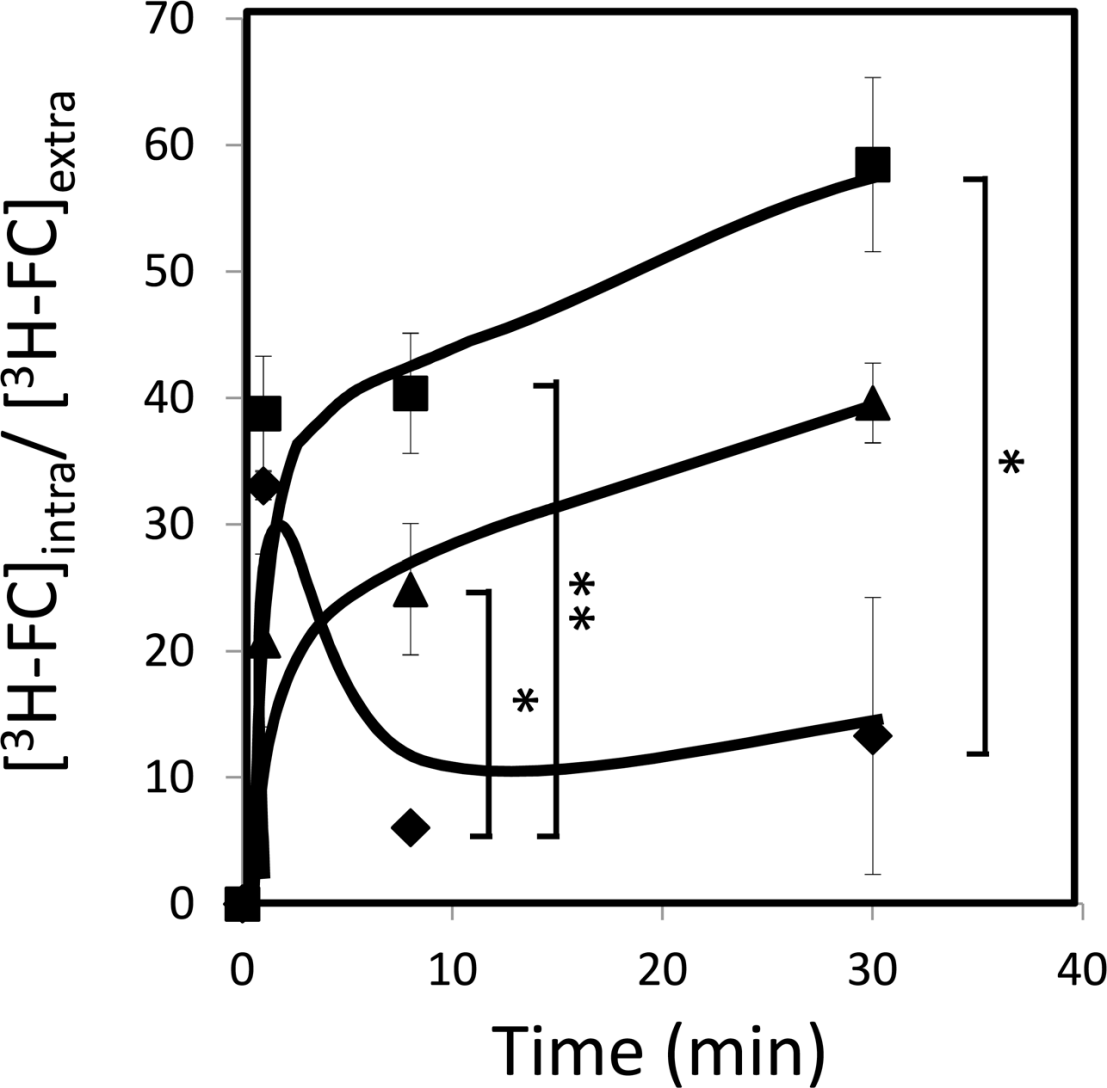
*CgFPS1* and *CgFPS2* expression decreases the accumulation of 5-flucytosine in *C*. *glabrata* cells. Time-course accumulation of [^3^H]-flucytosine in non-adapted KUE100 (♦), KUE100_*Δcgfps1* (■) and KUE100_*Δcgfps2* (▲) strains, during cultivation in BM liquid medium in the presence of 3mg/L of unlabelled 5-flucytosine. The indicated accumulation ratio values are averages of at least three independent experiments. Error bars represent the corresponding standard deviation. * P<0.05; **P<0.01.

To understand the role of CgFps1 and CgFps2 in the context of 5-flucytosine resistance, [^3^H]-flucytosine accumulation assays were carried out in the absence or presence of the encoding genes. Consistent with the observed susceptibility phenotypes, both *Δcgfps1* and *Δcgfps2* deletion mutants were found to accumulate around three-fold more radiolabelled 5-flucytosine that the corresponding parental KUE100 strain (Fig 5). Interestingly, the deletion of *CgFPS1* was found to exert a stronger effect in flucytosine accumulation than that of *CgFPS2*, which appears to be consistent with the effect of the deletion of each gene in the susceptibility to this antifungal drug. These results strongly suggests that CgFps1 and CgFps2 activities increase *C*. *glabrata* resistance towards 5-flucytosine by reducing its accumulation within yeast cells.

**Fig 6:**
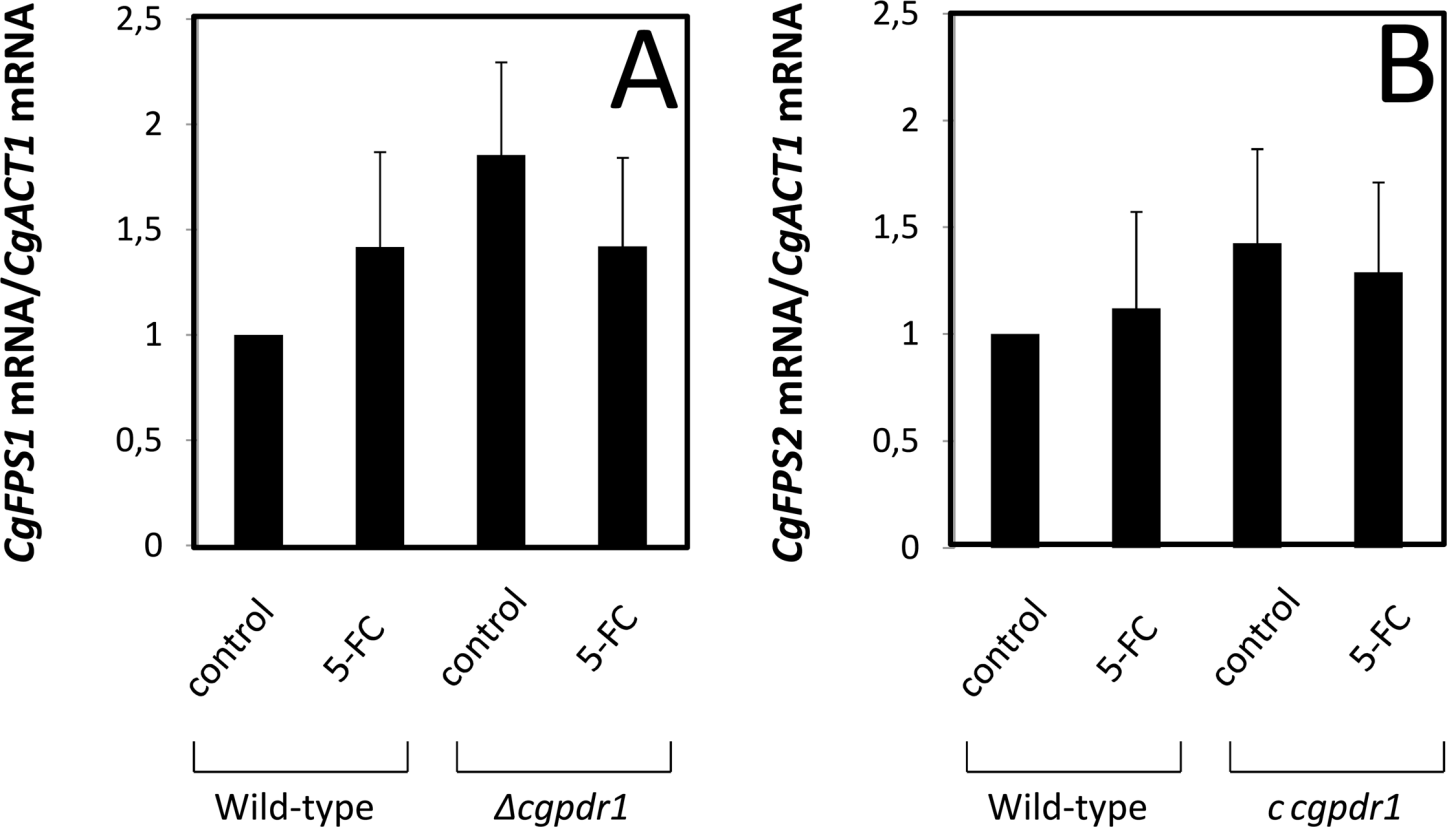
The expression of *CgFPS1* and *CgFPS2* is independent of 5-flucytosine exposure or *CgPDR1* deletion. Comparison of the variation of the *CgFPS1* (A) and *CgFPS2* (B) transcript levels in the 66032u *C*. *glabrata* wild-type strain and in the derived 66032u_*Δcgpdr1* deletion mutant, before (control) and after 1h of exposure to 3.5mg/L 5-flucytosine (5-FC). The presented transcript levels were obtained by quantitative RT-PCR and are relative *CgFPS1/CgACT1* or *CgFPS2/CgACT1* mRNA, relative to the values registered in the 66032 parental strain in control conditions. The indicated values are averages of at least three independent experiments. Error bars represent the corresponding standard deviation.

Since the drug resistance phenotype can be seen as a long term genetic stabilization of the normally transient drug response, the effect of *C*. *glabrata* cell exposure to inhibitory concentrations of 5-flucytosine in the transcriptional control of the *CgFPS1* and *CgFPS2* genes was evaluated. The transcript levels of *CgFPS1* and *CgFPS2* was seen to suffer no statistically significant change upon 1h of exposure of an un-adapted *Candida glabrata* population to 3.5 mg/l 5-flucytosine (Fig 6). Additionally, no effect of the deletion of the transcription CgPdr1, the major regulator of multidrug resistance in *C*. *glabrata*, could be observed, either in control or 5-flucytosine-stressed conditions (Fig 6).

## Discussion

In this work, the *S*. *cerevisiae* deletion mutant collection was used to gain new insights into the mechanisms of resistance and adaptation to 5-flucytosine, aiming the study of such mechanisms in the human pathogen *Candida glabrata*. Among the identified new mechanisms of resistance, three were selected for additional work in *C*. *glabrata*.

Within the nearly 200 determinants of 5-flucytosine resistance, around one third was found to be related to DNA repair, RNA metabolism and protein catabolism. These biological processes have a clear correlation with the mechanisms of action of 5-flucytosine. In both *S*. *cerevisiae* and *Candida* yeast cells, 5-flucytosine is converted to 5-fluorouracil, which is then converted into phosphorylated 5-fluorouridylic acid and incorporated into RNA, resulting in the perturbation of both RNA metabolism and in the disruption of protein synthesis [5]. 5-fluorouracil is alternatively converted into 5-fluorodeoxyuridine monophosphate, a potent inhibitor of thymidylate synthase, resulting in the inhibition of thymine synthesis and, thus, affecting DNA replication [5]. It is thus reasonable to assume that the DNA-, RNA- an protein-metabolism-related genes identified herein as conferring 5-flucytosine resistance are required to counteract or at least restrain the deleterious action of this antifungal drug. This is also consistent with the results from previous microarray analyses of the transcriptome-wide *S*. *cerevisiae* [11] or *Candida albicans* [30] response to 5-flucytosine, which showed that one third of the up-regulated genes are clustered into DNA repair, synthesis and replication and RNA metabolism.

Many of the genes identified in this study as conferring resistance to 5-flucytosine had not been previously linked to this phenomenon, not even in other chemogenomic analysis focused on 5-flucytosine resistance in *S*. *cerevisiae* [31] or *C*. *albicans* [32]. Surprisingly, only 19 of the 183 genes identified herein as 5-flucytosine resistance determinants had also been identified as such by Hillenmeyer and coworkers [31]. In the case of the chemogenomic analysis carried out for *C*. *albicans*, only 19 determinants of resistance to 5-flucytosine were identified [32], none of which coinciding with the genes identified in the present study. The differences in terms of the results obtained in each of these analyses are most likely related to the different experimental setup used. In both previous studies the relative effect of each gene deletion was analysed using a competition assay, followed by quantification of the frequency of each strain by deletion-tag microarray analysis [31][32], whereas in the present study the growth ability of each strain was analysed individually. Furthermore, the growth media in which the knockout collections were evaluated also varied from rich YPD medium [31], or RPMI medium [32] to minimal medium, in the current study. Additionally, in the case of the *C*. *albicans* study the knockout collection only covered one half of the genes in the genome [32].

Particularly noteworthy are the biological processes which are herein related for the first time to flucytosine resistance. Within these processes, the fact that five genes encoding arginine metabolic enzymes were found to confer 5-flucytosine resistance suggests that arginine itself may be required for 5-flucytosine resistance. Interestingly, L-arginine concentrations in human plasma [33] or vaginal fluid [34] in healthy individual reaches nearly 0.1mM. This may constitute a positive factor for 5-flucytosine resistance development in the human host. Although it is not clear what is the precise mechanism underlying the beneficial effect of arginine in 5-flucytosine challenged cells, the obtained results highlight the arginine uptake or biosynthetic pathway as a new interesting target for 5-flucytosine sensitization.

The requirement for cell wall and plasma membrane associated genes as mechanisms of resistance to 5-flucytosine is also highlighted in this study. Interestingly, the *ERG3* and *ERG4* genes, encoding ergosterol biosynthetic enzymes, were found to be determinants of 5-flucytosine resistance, suggesting that the sterol composition of the plasma membrane is a key factor in the tolerance to this antifungal drug. The fact that ergosterol biosynthesis is the target of two of the major families of antifungal drugs used in clinical practice, the azoles and the polyenes [6], which are usually prescribed in combination therapy with 5-flucytosine, raises concern on the possibility of cross-resistance development. This study further highlights the importance of cell wall proteins in yeast resistance to 5-flucytosine. Based on the obtained data, the changes undergone by the cell wall upon sudden 5-flucytosine challenge and after yeast adaptation to 5-flucytosine stress were studied using a lyticase susceptibility screening assay. It is remarkable to realize that just upon 30min of 5-flucytosine exposure, the *C*. *glabrata* cell wall becomes more susceptible to lyticase, suggesting that 5-flucytosine exerts a deleterious effect on the cell wall. Interestingly, shortly after contact with 5-flucytosine, a marked enlargement of *S*. *cerevisiae* and *C*. *albicans* cells was previously registered [35]. An electron microscope examination of *C*. *albicans* cells after exposure to the drug revealed characteristic changes, consisting of an enlarged nucleus and a thin cell wall [35]. This change in cell wall thickness may correlate with the observed increase in lyticase susceptibility. Also consistent with the harmful effect of 5-flucytosine in the cell wall is the observation that *C*. *glabrata* cells adapted to exponential growth in the presence of 5-flucytosine exhibit cell walls clearly more resistant to lyticase and Calcofluor White. The cell wall remodeling that underlies this observation is expected to depend on the cell wall related genes found to confer resistance to 5-flucytosine. Interestingly, the observed strengthening of the cell wall makes the 5-flucytosine adapted cells even more lyticase tolerant than non-stressed exponentially growing cells. The fact that 5-flucytosine has such an effect over the cell wall, a structure targeted directly by the newest class of antifungal drugs, the echinocandins, suggests that a combined therapy using echinocandins and 5-flucytosine may be a promising approach which, to the best of our knowledge, has not been attempted so far.

Finally, the aquaglyceroporin encoding gene *FPS1* was also identified as a determinant of yeast resistance to 5-flucytosine. The impact of this finding in *S*. *cerevisiae* was extended to the homologous *CgFPS1* and *CgFPS2* genes, from the pathogenic yeast *Candida glabrata*, which were found to contribute to 5-flucytosine resistance, by mediating a decrease in the accumulation of radiolabelled 5-flucytosine in *C*. *glabrata* cells. Fps aquaglyceroporins have been shown to work as facilitators of the diffusion of glycerol [13,20] but also, in what is believed to be a fortuitous effect, appear to facilitate the entrance or exit of a few other unrelated compounds. These include arsenite and antimonite [14], acetic acid [15], boron [16] and ethanol [17]. Whether this effect is direct or indirect appears to be arguable, especially since the deletion of Fps1 in *S*. *cerevisiae* has been demonstrated to have broad spectrum effects in cell physiology, including disturbance of the cell redox balance [18] and a decreased ergosterol concentration in the yeast plasma membrane [19]. Whatever the case is, in this study 5-flucytosine is added to the list of compounds whose intracellular accumulation depends on the Fps aquaglyceroporins, that may be working as diffusion channels of 5-flucytosine to the outer medium. Additionally, it was shown that *CgFPS1* or *CgFPS2* are not transcriptionally responsive to 5-flucytosine stress, which appears to be consistent with the notion that these genes are also not up-regulated under hyperosmotic stress [20]. It would be interesting to evaluate whether these genes suffer post-translational modifications upon 5-flucytosine exposure, as registered under osmotic stress [13,20], in the search for the signaling mechanisms that control 5-flucytosine accumulation.

Altogether, this chemogenomics study carried out in *S*. *cerevisiae*, brings a genome-wide scale to the understanding of 5-flucytosine resistance mechanisms. Some of the newly described phenomena in *S*. *cerevisiae*, as a model system, were tested in *C*. *glabrata*, as a pathogenic yeast. Although it will be interesting to verify in pathogenic yeasts some of the additional insights brought out by this analysis in *S*. *cerevisiae*, the current study highlights three new biological processes that affect 5-flucytosine resistance in *C*. *glabrata*: arginine homeostasis, cell wall remodeling and the aquaglyceroporins of the Fps family. These processes stand out as promising targets for the development of new 5-flucytosine chemosensitizers, which would expectedly allow for the use of decreased therapeutic dosages of 5-flucytosine, limiting the development of 5-flucytosine resistance which usually happens very fast, and, thus, enabling a more extensive (re)use of this antifungal drug.

## Supporting Information

S1 Fig Schematic representation of the homologous recombination procedure used for the deletion of *CgFPS1* and *CgFPS2* in the KUE100 *C*. *glabrata* strain, and of the selection of the primers used to confirm the deletion of these genes in their chromosomal locations. The results obtained for the confirmation of these constructs are shown below, using the parental strain as a control.
(TIF)

S1 Table Complete list of genes identified in this study as conferring resistance to 5-flucytosine, clustered into functional groups according to their description in the Saccharomyces Genome Database. (XLSX)

## References

[1] Fidel PL Jr., Vazquez JA, Sobel JD (1999) *Candida glabrata*: review of epidemiology, pathogenesis, and clinical disease with comparison to *C*. *albicans* . Clin Microbiol Rev 12: 80–96. 9880475 10.1128/cmr.12.1.80 PMC88907

[2] Mishra NN, Prasad T, Sharma N, Payasi A, Prasad R, Gupta DK, et al (2007) Pathogenicity and drug resistance in *Candida albicans* and other yeast species. A review. Acta Microbiol Immunol Hung 54: 201–235. 17896473 10.1556/AMicr.54.2007.3.1

[3] Ghannoum MA, Rice LB (1999) Antifungal agents: mode of action, mechanisms of resistance, and correlation of these mechanisms with bacterial resistance. Clin Microbiol Rev 12: 501–517. 10515900 10.1128/cmr.12.4.501 PMC88922

[4] Edlind TD, Katiyar SK (2010) Mutational analysis of flucytosine resistance in *Candida glabrata* . Antimicrob Agents Chemother 54: 4733–4738. 10.1128/AAC.00605-10 20823283 PMC2976130

[5] Hope WW, Tabernero L, Denning DW, Anderson MJ (2004) Molecular mechanisms of primary resistance to flucytosine in *Candida albicans* . Antimicrob Agents Chemother 48: 4377–4386. 15504867 10.1128/AAC.48.11.4377-4386.2004 PMC525410

[6] Espinel-Ingroff A (2008) Mechanisms of resistance to antifungal agents: yeasts and filamentous fungi. Rev Iberoam Micol 25: 101–106. 18473504 10.1016/s1130-1406(08)70027-5

[7] Vermes A, Guchelaar HJ, Dankert J (2000) Flucytosine: a review of its pharmacology, clinical indications, pharmacokinetics, toxicity and drug interactions. J Antimicrob Chemother 46: 171–179. 10933638 10.1093/jac/46.2.171

[8] Sanglard D, Odds FC (2002) Resistance of Candida species to antifungal agents: molecular mechanisms and clinical consequences. Lancet Infect Dis 2: 73–85. 11901654 10.1016/s1473-3099(02)00181-0

[9] Kontoyiannis DP, Lewis RE (2002) Antifungal drug resistance of pathogenic fungi. Lancet 359: 1135–1144. 11943280 10.1016/S0140-6736(02)08162-X

[10] Papon N, Noel T, Florent M, Gibot-Leclerc S, Jean D, Chastin C, et al (2007) Molecular mechanism of flucytosine resistance in *Candida lusitaniae*: contribution of the *FCY2*, *FCY1*, and *FUR1* genes to 5-fluorouracil and fluconazole cross-resistance. Antimicrob Agents Chemother 51: 369–371. 17060521 10.1128/AAC.00824-06 PMC1797687

[11] Zhang L, Zhang Y, Zhou Y, Zhao Y, Cheng J (2002) Expression profiling of the response of *Saccharomyces cerevisiae* to 5-fluorocytosine using a DNA microarray. Int J Antimicrob Agents 20: 444–450. 12458139 10.1016/s0924-8579(02)00201-7

[12] Schwarzmuller T, Ma B, Hiller E, Istel F, Tscherner M, Brunke S, et al (2014) Systematic phenotyping of a large-scale *Candida glabrata* deletion collection reveals novel antifungal tolerance genes. PLoS Pathog 10: e1004211 10.1371/journal.ppat.1004211 24945925 PMC4063973

[13] Támas MJ, Luyten K, Sutherland FC, Hernandez A, Albertyn J, Valadi H, et al (1999) Fps1p controls the accumulation and release of the compatible solute glycerol in yeast osmoregulation. Mol Microbiol 31: 1087–1104. 10096077 10.1046/j.1365-2958.1999.01248.x

[14] Wysocki R, Chery CC, Wawrzycka D, Van Hulle M, Cornelis R, Thevelein JM, et al (2001) The glycerol channel Fps1p mediates the uptake of arsenite and antimonite in *Saccharomyces cerevisiae* . Mol Microbiol 40: 1391–1401. 11442837 10.1046/j.1365-2958.2001.02485.x

[15] Mollapour M, Piper PW (2007) Hog1 mitogen-activated protein kinase phosphorylation targets the yeast Fps1 aquaglyceroporin for endocytosis, thereby rendering cells resistant to acetic acid. Mol Cell Biol 27: 6446–6456. 17620418 10.1128/MCB.02205-06 PMC2099610

[16] Nozawa A, Takano J, Kobayashi M, von Wiren N, Fujiwara T (2006) Roles of *BOR1*, *DUR3*, and *FPS1* in boron transport and tolerance in *Saccharomyces cerevisiae* . FEMS Microbiol Lett 262: 216–222. 16923078 10.1111/j.1574-6968.2006.00395.x

[17] Teixeira MC, Raposo LR, Mira NP, Lourenco AB, Sá-Correia I (2009) Genome-wide identification of *Saccharomyces cerevisiae* genes required for maximal tolerance to ethanol. Appl Environ Microbiol 75: 5761–5772. 10.1128/AEM.00845-09 19633105 PMC2747848

[18] Lourenço AB, Roque FC, Teixeira MC, Ascenso JR, Sá-Correia I (2013) Quantitative 1H-NMR-metabolomics reveals extensive metabolic reprogramming and the effect of the aquaglyceroporin Fps1 in ethanol-stressed yeast cells. PLoS One 8: e55439 10.1371/journal.pone.0055439 23408980 PMC3568136

[19] Toh TH, Kayingo G, van der Merwe MJ, Kilian SG, Hallsworth JE, Hohmann S, et al (2001) Implications of *FPS1* deletion and membrane ergosterol content for glycerol efflux from *Saccharomyces cerevisiae* . FEMS Yeast Res 1: 205–211. 12702345 10.1111/j.1567-1364.2001.tb00035.x

[20] Beese-Sims SE, Pan SJ, Lee J, Hwang-Wong E, Cormack BP, Levin DE (2012) Mutants in the *Candida glabrata* glycerol channels are sensitized to cell wall stress. Eukaryot Cell 11: 1512–1519. 10.1128/EC.00231-12 23087370 PMC3536289

[21] Ueno K, Uno J, Nakayama H, Sasamoto K, Mikami Y, Chibana H (2007) Development of a highly efficient gene targeting system induced by transient repression of *YKU80* expression in *Candida glabrata* . Eukaryot Cell 6: 1239–1247. 17513567 10.1128/EC.00414-06 PMC1951112

[22] Vermitsky JP, Edlind TD (2004) Azole resistance in *Candida glabrata*: coordinate upregulation of multidrug transporters and evidence for a Pdr1-like transcription factor. Antimicrob Agents Chemother 48: 3773–3781. 15388433 10.1128/AAC.48.10.3773-3781.2004 PMC521908

[23] Simões T, Teixeira MC, Fernandes AR, Sá-Correia I (2003) Adaptation of *Saccharomyces cerevisiae* to the herbicide 2,4-dichlorophenoxyacetic acid, mediated by Msn2p- and Msn4p-regulated genes: important role of *SPI1* . Appl Environ Microbiol 69: 4019–4028. 12839777 10.1128/AEM.69.7.4019-4028.2003 PMC165130

[24] Ueno K, Matsumoto Y, Uno J, Sasamoto K, Sekimizu K, Kinjo Y, et al (2011) Intestinal resident yeast *Candida glabrata* requires Cyb2p-mediated lactate assimilation to adapt in mouse intestine. PLoS One 6: e24759 10.1371/journal.pone.0024759 21931845 PMC3170380

[25] Jansen G, Wu C, Schade B, Thomas DY, Whiteway M (2005) Drag&Drop cloning in yeast. Gene 344: 43–51. 15656971 10.1016/j.gene.2004.10.016

[26] Costa C, Henriques A, Pires C, Nunes J, Ohno M, Chibana H, et al (2013) The dual role of *Candida glabrata* drug:H+ antiporter CgAqr1 (ORF *CAGL0J09944g*) in antifungal drug and acetic acid resistance. Front Microbiol 4: 170 10.3389/fmicb.2013.00170 23805133 PMC3693063

[27] Costa C, Nunes J, Henriques A, Mira NP, Nakayama H, Chibana H, et al (2014) *Candida glabrata* drug:H+ antiporter CgTpo3 (ORF *CAGL0I10384g*): role in azole drug resistance and polyamine homeostasis. J Antimicrob Chemother 69: 1767–1776. 10.1093/jac/dku044 24576949

[28] Costa C, Pires C, Cabrito TR, Renaudin A, Ohno M, Chibana H, et al (2013) *Candida glabrata* Drug:H+ Antiporter CgQdr2 Confers Imidazole Drug Resistance, Being Activated by Transcription Factor CgPdr1. Antimicrob Agents Chemother 57: 3159–3167. 10.1128/AAC.00811-12 23629708 PMC3697362

[29] Rosa MF, Sá-Correia I (1996) Intracellular acidification does not account for inhibition of *Saccharomyces cerevisiae* growth in the presence of ethanol. FEMS Microbiology Letters 135: 271–274. 8595868 10.1111/j.1574-6968.1996.tb08000.x

[30] Liu TT, Lee RE, Barker KS, Wei L, Homayouni R, Rogers PD (2005) Genome-wide expression profiling of the response to azole, polyene, echinocandin, and pyrimidine antifungal agents in *Candida albicans* . Antimicrob Agents Chemother 49: 2226–2236. 15917516 10.1128/AAC.49.6.2226-2236.2005 PMC1140538

[31] Hillenmeyer ME, Fung E, Wildenhain J, Pierce SE, Hoon S, Lee W, et al (2008) The chemical genomic portrait of yeast: uncovering a phenotype for all genes. Science 320: 362–365. 10.1126/science.1150021 18420932 PMC2794835

[32] Xu D, Jiang B, Ketela T, Lemieux S, Veillette K, Martel N, et al (2007) Genome-wide fitness test and mechanism-of-action studies of inhibitory compounds in *Candida albicans* . PLoS Pathog 3: e92 17604452 10.1371/journal.ppat.0030092 PMC1904411

[33] Armengou A, Hurtado O, Leira R, Obon M, Pascual C, Moro MA, et al (2003) L-arginine levels in blood as a marker of nitric oxide-mediated brain damage in acute stroke: a clinical and experimental study. J Cereb Blood Flow Metab 23: 978–984. 12902842 10.1097/01.WCB.0000080651.64357.C6

[34] Gregoire AT, Lang WR, Ward K (1959) The qualitative identification of free amino acids in human vaginal fluid. Ann N Y Acad Sci 83: 185–188. 13851688 10.1111/j.1749-6632.1960.tb40891.x

[35] Arai T, Mikami Y, Yokoyama K, Kawata T, Masuda K (1977) Morphological changes in yeasts as a result of the action of 5-fluorocytosine. Antimicrob Agents Chemother 12: 255–260. 332075 10.1128/aac.12.2.255 PMC429894

