# Supplementary figures and images for: New Mechanisms of Flucytosine Resistance in C. glabrata Unveiled by a Chemogenomics Analysis in S. cerevisiae
Source: PLoS One. 2015 Aug 12;10(8):e0135110. doi: 10.1371/journal.pone.0135110 (PMC4534419; doi:10.1371/journal.pone.0135110)

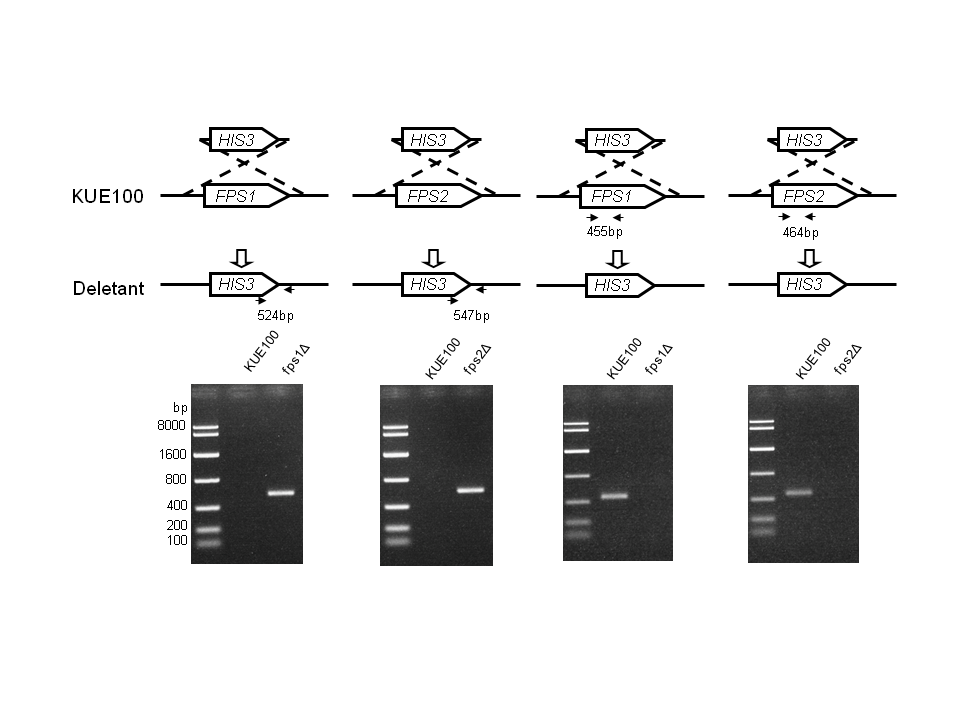

Supplement: S1 Fig — The results obtained for the confirmation of these constructs are shown below, using the parental strain as a control. (TIF) [file pone.0135110.s001.tif]
